# Views of the Midlands on Worcester Infirmary

**Published:** 1913-09-06

**Authors:** 


### VIEWS OF THE MIDLANDS ON WORCESTER INFIRMARY.

The, literally, unprecedented condition of hospital affairs at Worcester, where the *Worcester Times* has admitted that the local dignitaries, from the Bishop, Dr. Yeatman-Biggs, to the Mayor, have done their best, and "failed to rouse Worcestershire people to a more adequate recognition of the claims and needs of the infirmary," has startled the whole Midlands into amazed attention. For having achieved this the *Worcester Times* deserves and must have full credit, since if the Worcestershire people cannot be moved by their own authorities and reformed from within, the only alternative is to reform them from without. Birmingham has been the first to call attention to the supineness of which the Worcester newspaper has to complain, and in a thoughtful article the *Birmingham Post* last week recalls the disastrous story of the continuous sale of the hospital's funded property, the issue of the Bishop's appeal, the failure of which resulted from the city's indifference to it, the feeble report of a few weeks ago, and the shifts of administrative bankruptcy, which were suggested in it. The repetition of this story throughout the country should be enough to concentrate hospital and public opinion in such a way as to rouse the Worcester citizens to remove the intolerable stigma which at present rests upon them. The proof that this first step has been taken will be the re-forming of the committee, since members must be found to start afresh who are not directly responsible for the policy of despair which has prevailed hitherto, and when that has been done the infirmary's future should be on the way to rehabilitation. From Worcester to Birmingham the story of the past has spread, and when it is repeated throughout the country, as it will be, since other cathedral cities are already reading it with amazement, the Worcester citizens will realise that to be moribund is not publicly approved. Then the necessary money will, we believe, be forthcoming, and when it is, it will not lessen our thankfulness that our share in stimulating public opinion led us to be called "the enemy" by certain flustered people who were not, at the time, more than half awakened from their sleep.

